# Genotyping and macrolide-resistant mutation of *Bordetella pertussis* in East and South-East Asia

**DOI:** 10.1016/j.jgar.2022.10.007

**Published:** 2022-12

**Authors:** Kentaro Koide, ShuMan Yao, Chuen‑Sheue Chiang, Phung Thi Bich Thuy, Do Thi Thuy Nga, Do Thu Huong, Tran Minh Dien, Ork Vichit, Yong Vutthikol, Siphan Sovannara, Chham Samnang, Ikuyo Takayama, Akira Ainai, Noriko Nakajima, Nao Otsuka, Kazunari Kamachi, Akihiko Saitoh

**Affiliations:** a Department of Bacteriology II*,* National Institute of Infectious Diseases*,* Tokyo*,* Japan; b Centre for Diagnostics and Vaccine Development, Centres for Disease Control, Taipei, Taiwan; c Department of Molecular Biology for Infectious Disease, Vietnam National Children's Hospital, Hanoi, Vietnam; d Division of General Internal Medicine, Centre for Tropical Diseases, Vietnam National Children's Hospital, Hanoi, Vietnam; e Surgical Intensive Care Unit, Vietnam National Children's Hospital, Hanoi, Vietnam; f National Immunization Program, Ministry of Health, Cambodia; g Vaccine-Preventable Diseases and Immunization, World Health Organization, Cambodia; h Research Centre for Influenza and Respiratory Viruses, National Institute of Infectious Diseases, Tokyo, Japan; i Department of Pathology, National Institute of Infectious Diseases, Tokyo, Japan; j Department of Pediatrics, Niigata University Graduate School of Medical and Dental Sciences, Niigata, Japan

**Keywords:** *Bordetella pertussis*, East and Southeast Asia, Macrolide-resistant A2047G mutation, 23S rRNA, Multilocus variable-number tandem-repeat analysis, Whole-genome sequencing

## Abstract

**Objectives:**

Macrolide-resistant *Bordetella pertussis* (MRBP) has been emerging and prevailing in mainland China since 2011. In this study, we aimed to investigate the genotype and macrolide resistance of circulating *B. pertussis* in East and Southeast Asia using genetic analyses.

**Methods:**

A total of 302 DNA extracts from clinical specimens and isolates from 2010 to 2020 were analyzed: 145 from Vietnam, 76 from Cambodia, 48 from Taiwan, and 33 from Japan. Genotypes were determined by multilocus variable-number tandem-repeat analysis (MLVA). Macrolide-resistant A2047G mutation in *B. pertussis* 23S rRNA was investigated using the duplex Cycleave real-time polymerase chain reaction (PCR) assay. Whole-genome sequencing was performed on two MRBP isolates that were identified for the first time in Taiwan.

**Results:**

Overall, 286 DNA extracts (95%) generated a complete MLVA genotype and 283 DNA extracts (94%) yielded a complete result for the A2047G mutation analysis. The A2047G mutation was detected in 18 DNA extracts: fourteen from Vietnam, one from Cambodia, two from Taiwan, and one from Japan. Most of them (78%) showed the genotypes MT104 and MT195, which have previously been reported in Chinese MRBP isolates. Further, the Taiwanese MRBP isolates were classified into the MT104 clade of Chinese MRBP isolates.

**Conclusion:**

After MRBP emerged and spread in mainland China, it may have spread to East and Southeast Asia in the 2010s. Continued surveillance targeting the A2047G mutation of MRBP is needed to prevent further spread of this emerging pathogen.

## Highlights

• Molecular surveillance of macrolide-resistant *Bordetella pertussis* (MRBP) was performed in East and Southeast Asia.
• MBRP was detected in Vietnam, Cambodia, Taiwan, and Japan.
• Most MRBP had the same MT104 and MT195 genotypes as Chinese MRBP.
• The first MRBP isolates from Taiwan belonged to the MT104 cluster of Chinese MRBP.

## 1 Introduction

Pertussis (whooping cough), caused by the Gram-negative bacterium *Bordetella pertussis*, manifests as a severe acute respiratory disease among neonates, infants, and children, and a persistent cough among adolescents and adults. Vaccination is an effective method for preventing and controlling pertussis, but pertussis vaccines do not provide lifelong immunity [1,2]. Pertussis persists despite high vaccination coverage in many countries. Macrolides, such as azithromycin, clarythromycin, and erythromycin, are frequently used to treat and prevent pertussis; however, since 2011, macrolide-resistant *B. pertussis* (MRBP) has been emerging and prevailing in mainland China [3], [4], [5], [6]. Two MRBP isolates were first detected in 2011 in Shandong province, China [7], and then MRBP was isolated with increasing frequency (57.5%–91.9%) in China between 2011 and 2020. Recently, MRBP has been detected in Vietnam (located in Southeast Asia) and Japan (in East Asia) [8,9].

MRBP isolates from China have a homogeneous A2047G mutation in each of the three copies of the 23S rRNA gene, which is associated with macrolide resistance [5,10,11]. The MRBP isolates exhibited high minimum inhibitory concentrations (MICs) of >256 µg/mL for macrolides. Further, most Chinese MRBP isolates have unique genotypes of MT55, MT104, and MT195, which are closely related based on multilocus variable-number tandem-repeat analysis (MLVA) [4,12,13]. Some MRBP isolates have minor MLVA genotypes such as MT107 and MT340, which branched from the three major genotypes. A significant correlation exists between macrolide resistance and MLVA genotypes in Chinese *B. pertussis* isolates. Whole-genome analysis based on single-nucleotide polymorphisms (SNPs) also shows that Chinese MRBP isolates can be classified into three subclades relating to MLVA genotypes MT55, MT104, and MT195 [13], which are estimated to have diverged in the late 2000s [14]. Previous studies suggest that MRBP may be identifiable by MLVA genotyping.

Culture of *B. pertussis* has limited sensitivity among previously vaccinated persons, older children, adolescents, and adults. Moreover, culturing has the disadvantage of requiring isolation media specifically for *B. pertussis* (e.g., Bordet-Gengou agar). Therefore, MLVA genotyping is performed not only on the bacterial isolates, but also on DNA extracts from clinical specimens [15], [16], [17], [18]. Likewise, a macrolide-resistant A2047G mutation in the *B. pertussis* 23S rRNA gene can also be investigated directly by genetic analysis using clinical specimens [8,11,19]. A previous study demonstrated the emergence of MRBP carrying the A2047G mutation in Vietnam during 2016 to 2017 that had the same or similar MLVA genotypes (MT104 and its variants) to a Chinese MRBP strain [8]. In Cambodia and Japan, the major MLVA genotypes of MRBP (MT55, MT104, and MT195) were not detected among *B. pertussis* isolates between 2005 and 2016 [18,20]. However, the MLVA genotypes and macrolide-resistance profiles of *B. pertussis* in these countries have not been reported in recent years.

In this study, we investigated the MLVA genotypes and macrolide resistance of *B. pertussis* using clinical specimens and isolates from Vietnam, Cambodia, Taiwan, and Japan in order to survey MRBP in East and Southeast Asia. In Taiwan, we investigated isolates collected since 2010 because no information has been reported on the MLVA genotype to date. Further, we performed whole-genome sequencing (WGS) of two MRBP isolates identified for the first time in Taiwan, and assessed their genetic relationships with Chinese MRBP using public sequence databases.

## 2 Materials and methods

### 2.1 Clinical specimens and isolates

One hundred and thirty-nine nasal washes and six *B. pertussis* isolates were collected from pediatric pertussis patients as part of routine clinical practice in Vietnam National Children's Hospital (VNCH) during 2017 to 2020, and 76 nasopharyngeal swabs were collected nationwide in Cambodia during the same time period (Supplementary Fig. S1). DNA was extracted using the MagMAX CORE Nucleic Acid Purification Kit (Thermo Fisher) for the nasal washes and QIAamp DNA Micro Kit (QIAGEN) for the nasopharyngeal swabs. Forty-eight *B. pertussis* isolates were collected in Taiwan from 2010 to 2019, and 33 were collected in Japan from 2017 to 2019. The isolates were cultured on Bordet-Gengou agar or Bordetella CFDN agar plate (Nikken Bio Medical Laboratory, Japan), and then bacterial DNA was extracted. Detailed information on the clinical specimens and isolates is provided in Supplementary Table S1.

### 2.2 MLVA genotyping

Direct MLVA genotyping was performed on DNA extracted from clinical specimens by nested-multiplex polymerase chain reaction (PCR), with minor modifications [18]. Briefly, the first PCR was performed in a 25 µL reaction volume containing 2 µL of DNA sample, 0.32 mM dNTPs, 0.4 U of KOD-FX Neo DNA polymerase (TOYOBO, Japan), and 80 nM of each primer. The PCR conditions were 94° C for 2 min, followed by 40 cycles of 98° C for 10 s and 68° C for 1 min, and a final extension of 72° C for 5 min. The PCR products were treated with exonuclease I and then subjected to a second PCR. The MLVA genotyping of isolates was performed using a single multiplex PCR assay [21].

### 2.3 Analysis of macrolide resistance mutation

The macrolide-resistant A2047G mutation in *B. pertussis* 23S rRNA was analyzed using the duplex Cycleave real-time PCR assay (Takara Bio Inc., Japan), as described previously [8].

### 2.4 Whole-genome sequencing

Genomic DNA was purified from the Taiwanese MRBP isolates, 10015 and 10120, by the NucleoSpin Tissue kit (Macherey-Nagel, Germany), and the samples were sequenced on the Illumina NovaSeq 6000 platform with 150-bp paired-end reads performed by Novogene (Beijing, China). The average coverage depth of the sequencing was >300x for both isolates. The sequence data were submitted to the DDBJ Sequence Read Archive (DRA) (accession nos. DRR342996 and DRR342997).

### 2.5 SNP calling and phylogenetic analysis

Taiwanese MRBP isolates were compared with 46 Chinese MRBP isolates collected during 2012 to 2015 (Supplementary Table S2). Public genome data (Illumina short-reads) of the Chinese MRBP isolates were obtained from the European Sequence Read Archives. Sequence data were each assembled de novo by using CLC Genomic Workbench version 20.0.4 (CLC Bio, Denmark). In order to call SNPs, contigs were mapped to the reference genome sequence of MRBP isolate BP616 (GenBank accession no. AP024746.1) using CSI Phylogeny 1.4 [22]. The accuracy of SNP calls was manually inspected for mapped reads to the reference genome using the CLC Genomic Workbench, and a total of 163 high-quality SNPs were identified (Supplementary Table S2). A maximum parsimony tree was constructed based on the 163 SNPs using MEGA10 version 10.1.8 [23].

### 2.6 Data analysis

Simpson's diversity index was calculated using an online tool (http://www.comparingpartitions.info/). Minimum spanning trees were generated based on the MLVA data using FPQuest software (Bio-Rad).

## 3 Results

### 3.1 MLVA genotypes of *B. pertussis*

**Fig. 1:**
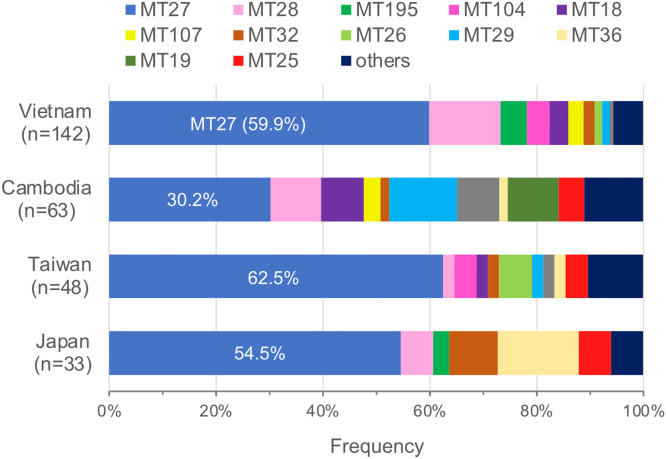
Genotypic diversity of *Bordetella pertussis* population in Vietnam, Cambodia, Taiwan, and Japan. The frequencies of multilocus variable-number tandem-repeat analysis genotypes are shown.

DNA extracts from clinical specimens and isolates were analyzed by MLVA genotyping. Of 145 Vietnamese and 76 Cambodian DNA samples, 142 and 63, respectively, yielded complete MLVA profiles, and the remainder yielded incomplete profiles (Supplementary Table S1). In contrast, all DNA samples from Taiwanese and Japanese isolates yielded complete MLVA profiles. The numbers of distinct MLVA genotypes (MTs) identified were 17, 16, 14, and 8 in Vietnam, Cambodia, Taiwan, and Japan, respectively (Table 1). The genotypic diversity was higher in Cambodia than in Vietnam, Taiwan, and Japan (diversity index, 0.870 vs. 0.609–0.682, *P* < 0.05). Further, the global MT27 strain was identified in all four study countries, but its frequency was lower in Cambodia than in Vietnam, Taiwan, and Japan (30.2% vs. 54.5–62.5%) (Fig. 1).

**Table 1:** MLVA genotypes of *Bordetella pertussis* in Southeast and East Asia

Area	Country	Year	Type of samples	No. of samples	No. of complete MLVA profiles	MLVA genotypes	Simpson's diversity index (95% CI)
South-East Asia	Vietnam	2017–2020	Nasal wash and isolate	145	142	MT16, MT18, MT26, MT27, MT28, MT29, MT32, MT33, MT96, MT104, MT106, MT107, MT113, MT125, MT195, Novel type B and D	0.621 (0.532–0.709)
	Cambodia	2017–2020	Nasopharyngeal swab	76	63	MT16, MT18, MT19, MT25, MT27, MT28, MT29, MT32, MT36, MT83, MT95, MT107, MT158, MT168, Novel type E and G	0.870 (0.815–0.924)
East Asia	Taiwan	2010–2019	Isolate	48	48	MT16, MT18, MT25, MT26, MT27, MT28, MT29, MT32, MT34, MT36, MT104, MT116, MT158, Novel type F	0.609 (0.444–0.774)
	Japan	2017–2019	Isolate	33	33	MT25, MT27, MT28, MT32, MT34, MT36, MT157, MT195	0.682 (0.523–0.841)

CI, confidence interval; MLVA, multilocus variable-number tandem-repeat analysis.

### 3.2 Macrolide resistance mutation in *B. pertussis*

DNA extracts from clinical specimens and isolates were analyzed using duplex Cycleave real-time PCR. Of 145 Vietnamese DNA samples, 131 yielded a complete result (with or without the A2047G mutation). Among them, 14 (10.7%) with MLVA genotypes MT104, MT107, MT195, and novel type B (defined in our previous study) were positive for the A2047G mutation (Table 2) [8]. Of 71 Cambodian DNA samples that yielded a complete result, one (1.4%), with the MLVA genotype MT28, was positive for the A2047G mutation. Of 48 Taiwanese DNA samples, two (4.2%), with the MLVA genotype MT104, were positive for the A2047G mutation. Of 33 Japanese DNA samples, one (3.0%), with the MLVA genotype MT195, was positive for the A2047G mutation. The Japanese MRBP isolate BP616 (alias 2018-56) had been genotyped previously [9], and the result was the same as that obtained in this study.

**Table 2:** Macrolide-resistant A2047G mutation in *Bordetella pertussis* in Southeast and East Asia

Area	Country	No. of samples	No. of samples with complete results	No. of samples without the A2047G mutation	No. of samples with the A2047G mutation (%)	MLVA genotypes with the A2047G mutation
South-East Asia	Vietnam	145	131	117	14 (10.7)	MT104, MT107, MT195, Novel type B
	Cambodia	76	71	70	1 (1.4)	MT28
East Asia	Taiwan	48	48	46	2 (4.2)	MT104
	Japan	33	33	32	1 (3.0)	MT195

MLVA, multilocus variable-number tandem-repeat analysis.

The two Taiwanese isolates carrying the A2047G mutation (isolate IDs 10015 and 10120) were confirmed as MRBP by E-test (bioMérieux, France). The isolates had MICs >256 µg/mL for the macrolides, azithromycin, erythromycin, and clarithromycin. The MRBP isolates were identified for the first time in Taiwan.

### 3.3 The A2047G mutation among *B. pertussis* populations

**Fig. 2:**
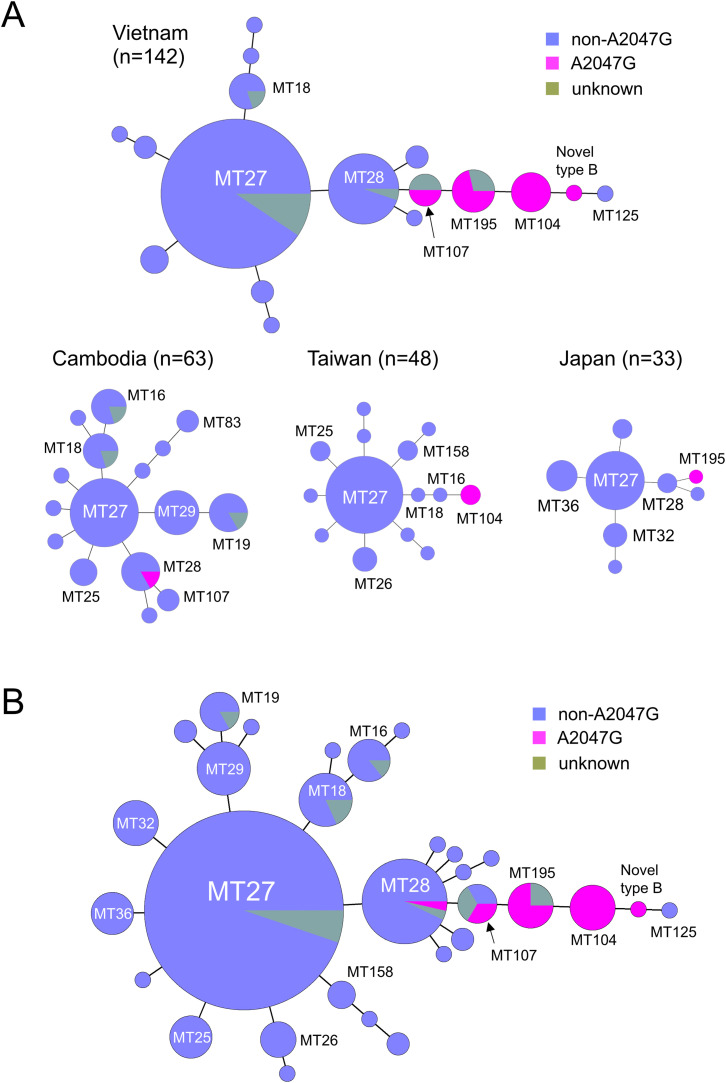
Minimum spanning trees revealing the genotypic diversity of *Bordetella pertussis* populations. (A) The populations in Vietnam, Cambodia, Taiwan, and Japan. (B) Combined populations of Vietnam, Cambodia, Taiwan, and Japan. The colors represent presence or absence of the A2047G macrolide resistance mutation. Each circle within a tree represents a unique MT, and the sizes of circles are representative of the number of clinical specimens or isolates in each group. MT, MLVA genotype.

Figure 2A represents minimum spanning trees (MST) that revealed the A2047G mutation among *B. pertussis* populations in Vietnam, Cambodia, Taiwan, and Japan. In Vietnam, the A2047G mutation was present in four MTs (MT104, MT107, MT195, and novel type B), and these MTs were closely linked to each other. In Cambodia, the A2047G mutation was detected in MT28, but five of six DNA samples of MT28 did not have the A2047G mutation. In Taiwan, the A2047G mutation in MT104 branched from MT16, whereas in Japan, it was in MT195 branched from MT28. Figure 2B shows an MST that combined the *B. pertussis* populations of Vietnam, Cambodia, Taiwan, and Japan. The five MTs with the A2047G mutation (MT28, MT104, MT107, MT195, and novel type B) were closely related to each other. Some samples of MT28 and MT107 did not have the A2047G mutation, but all samples of MT104 and MT195 had the A2047G mutation. MT125, which branched from novel type B, did not have the A2047G mutation.

### 3.4 Detection of the A2047G mutation by year

A total of 18 DNA samples were positive for the A2047G mutation. Among them, 16 DNA samples were collected from 2017 to 2020 in Vietnam, Cambodia, and Japan, and the remainder were collected from 2011 to 2012 in Taiwan (Table 3). Notably, none of the 30 Taiwanese DNA samples collected from 2013 to 2019 had the A2047G mutation. There was a temporal difference in the emergence of MRBP between Taiwan and the other three Asian countries studied.

**Table 3:** Summary of A2047G-positive samples by collection year in Southeast and East Asia

Area	Country	No. of samples with the A2047G mutation/No. of samples without the A2047G mutation							
		2010	2011	2012	2013–2016	2017	2018	2019	2020
South-East Asia	Vietnam					8/69		6/37	0/11
	Cambodia					0/17	0/21	0/20	1/12
East Asia	Taiwan	0/5	1/8	1/3	0/19	0/4	0/6	0/1	
	Japan					0/3	1/14	0/15	

### 3.5 Whole-genome phylogenetic analysis of Taiwanese MRBP isolates

**Fig. 3:**
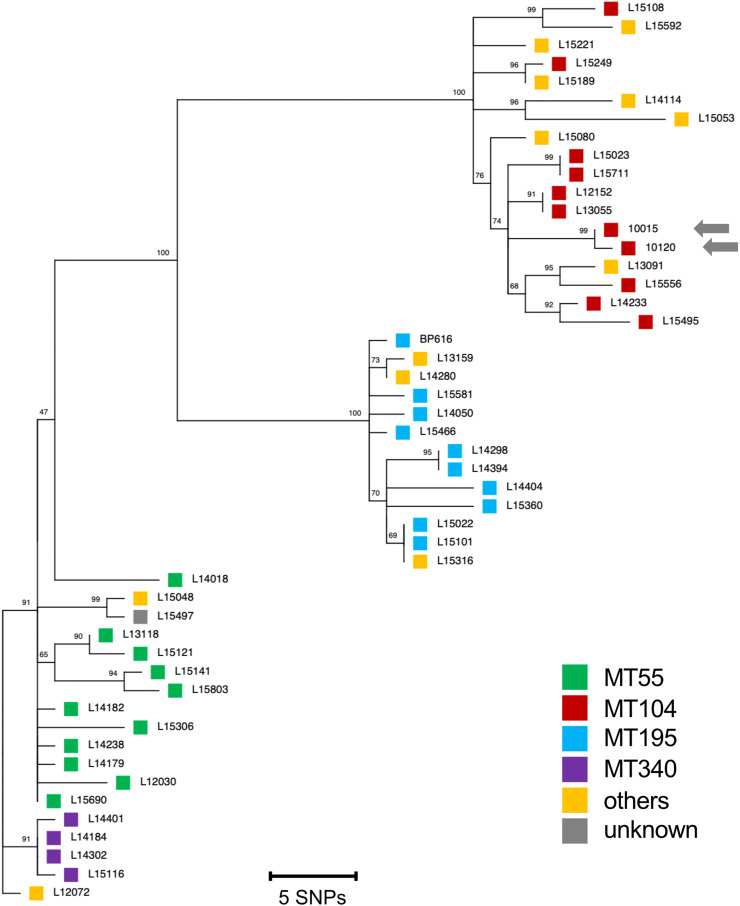
Phylogenetic relationship of the Taiwanese macrolide-resistant *Bordetella pertussis* (MRBP) isolates, 10015 and 10120, with 46 Chinese MRBP isolates. The maximum parsimony tree was constructed based on 163 single-nucleotide polymorphisms (SNPs) with 1000 bootstrap replicates. The Taiwanese MRBP isolates belong to the MT104 clade of Chinese MRBP isolates (indicated by arrows). The Japanese MRBP isolate, BP616, was used as a reference genome and is included in the phylogenetic tree.

The Taiwanese MRBP isolates, 10015 and 10120, were compared with 46 Chinese MRBP isolates using 163 whole-genome SNPs. As shown in Fig. 3, 46 Chinese MRBP isolates were classified into three major clades, and the Taiwanese MRBP isolates (MLVA genotype, MT104) belonged to the MT104 clade. The Taiwanese MRBP isolates were most closely related to the Chinese MRBP isolates, L12152 and L13055, collected in Shannxi province during 2012 to 2013 [13], with 7 to 8 SNP differences. The two Taiwanese MRBP isolates were collected in different districts but had only one SNP difference between their non-coding regions (isolate 10015 from Taoyuan City in 2011 and isolate 10120 from Hualien County in 2012).

## 4 Discussion

In this study, we investigated genotypes and macrolide resistance of *B. pertussis* in East and Southeast Asia using clinical specimens and isolates. The data showed that MRBP carrying the A2047G mutation occurred sporadically in Cambodia, Taiwan, and Japan, and frequently in Vietnam. The MRBP strains were of five closely related genotypes, and most genotypes were the same as those of the Chinese MRBP strains. The whole-genome analysis also showed that the first two MRBP isolates from Taiwan belonged to the MT104 clade of the Chinese MRBP lineage. To our knowledge, this is the most recent study (aside from those conducted in mainland China) investigating the emergence of MRBP in East and Southeast Asia.

The genetic analyses demonstrated that five MTs (MT28, MT104, MT107, MT195, and novel type B) were associated with the A2047G mutation in *B. pertussis* 23S rRNA. Of the five MTs, four had been identified previously: MT104, MT107, and MT195 in China, and novel type B in Vietnam [8,12,13]. MT28-MRBP was detected in Cambodia and linked to MT107 on the minimum spanning tree (Fig. 2B). MT28-MRBP was detected in only one DNA sample from Cambodia, suggesting that MT28-MRBP is rare among currently circulating MRBP strains in the four countries studied. In mainland China, MRBP was frequently isolated between 2011 and 2020, and its major genotypes (MT55, MT104, and MT195) have remained the same [4]. Combined with this previous research, our data show that the common MRBP strains have emerged in East and Southeast Asia, except for Cambodia.

In 2022, the emergence of novel MRBP strains carrying *ptxP3* allele (MT28-MRBP and MT27-MRBP) was reported from mainland China [24]. In general, MRBP strains carry the *B. pertussis* virulence-associated allelic genes, *ptxP1* and *fhaB3* [8,13,14]. In this study, we further characterized the allelic genes of four MT28 DNA samples from Cambodia (including the MT28-MRBP sample) by direct DNA sequencing, and detected *ptxP3* and non-*fhaB3* (i.e. *fhaB1* or *fhaB2*) alleles in all DNA samples. MT28-MRBP has different alleles from major MRBP isolates previously identified, suggesting that MT28-MRBP, which emerged in Cambodia, is unrelated to MRBP strains circulating in Vietnam. However, its relation to a Chinese MT28-MRBP is unknown. *Bordetella pertussis* carrying *ptxP3* allele is currently epidemic worldwide; thus, MRBP carrying *ptxP3* may increase globally in the future. Continuous surveillance of MT28-MRBP is required in Cambodia.

Previously, we reported the emergence of MRBP in Vietnam during 2016 to 2017 [8]. During the study period, MT104-MRBP was the predominant MRBP strain, and its variants (novel types A and B) were minor MRBP strains. In this study, two MTs (MT107 and MT195) were newly detected types of MRBP in Vietnam, showing that there has been an increase in the genotypic diversity of recent MRBP strains. One possible explanation for the increased diversity is that MT107- and MT195-MRBP strains were imported to Vietnam. Another possible explanation is a change in genotypes from MT104-MRBP to MT107- and MT195-MRBP. Genotype MT195 is a single-locus variant (SLV) of MT104, differing in only one of six variable-number tandem-repeat loci. Genotype MT107 is also an SLV of MT195. The reason for the increased diversity is currently unknown but may result from widespread antibiotic use in Vietnam.

In Taiwan, MRBP was not detected during 2003 to 2007 [25]. In this study, we identified two MRBP isolates from different districts in Taiwan in 2011 and 2012. Notably, the Taiwanese MRBP isolates were collected during the same period as the first isolation of MRBP in China [7]. The Taiwanese MRBP isolates (10015 and 10120) had the genotype MT104 and belonged to the MT104 clade of Chinese MRBP (Fig. 3). The Taiwanese MRBP isolates had only one SNP difference between them, and had 7 to 15 SNP differences (mean: 10 SNPs) compared with nine Chinese MT104-MRBP isolates. Similarly, the Chinese MT104-MRBP isolates had 0 to 16 SNP differences (mean: 8.7 SNPs) among them. The SNP difference between the Taiwanese and Chinese MT104-MRBP was within the range of that of the Chinese MRBP. This suggests that the Taiwanese MRBP isolates were genetically related to the Chinese MT104-MRBP.

In this study, we showed the sporadic emergence of MRBP in Cambodia, Taiwan, and Japan (Table 3). In contrast, in Vietnam, MRBP was present in 10 to 14% of samples in 2017 and 2019. MRBP was first detected in 2016 in Vietnam [8], suggesting that this resistant bacterium has a longstanding presence in Vietnam, as in mainland China. Macrolides are commonly used to treat respiratory infection and are available without prescription in both urban and rural areas in Vietnam [26]. Overuse and inappropriate use of macrolides may be associated with the continuous emergence of MRBP.

Macrolides are the first-choice drugs for pertussis infection, but they have a low bactericidal activity against MRBP [27]. MRBP epidemics lead to an increased risk of secondary infections among susceptible children. In fact, in China, a pertussis outbreak due to MT195-MRBP occurred in a primary school in 2016 [28]. An outbreak of MRBP may significantly increase its spread outside the epidemic area. The results of this study suggest that after MRBP emerged and spread in China, it may have spread to East and Southeast Asia. As a new MRBP (*ptxP3* strain) was also detected in Cambodia, continued surveillance of MRBP is needed to prevent further spread in Asia. We propose that the A2047G mutation is the most suitable genomic marker for rapid genetic screening of MRBP.

## Funding

This study was supported by the Japan Agency for Medical Research and Development (AMED) grants JP21fk0108139 and JP21fk0108082.

## Competing interests

None declared

## Ethical approval

Ethical approval for specimen collection in Vietnam was obtained from the Human Ethics Committee of VNCH and Niigata University (approval number 2019-0340). The specimens of Cambodia were collected for diagnostic and surveillance purposes as part of Cambodia's national immunization program.

## Appendix Supplementary materials

Image, application 1

Image, application 2
