# Supplementary figures and images for: Genotyping and macrolide-resistant mutation of Bordetella pertussis in East and South-East Asia
Source: J Glob Antimicrob Resist. 2022 Dec;31:263–9. doi: 10.1016/j.jgar.2022.10.007 (PMC9750937; doi:10.1016/j.jgar.2022.10.007)

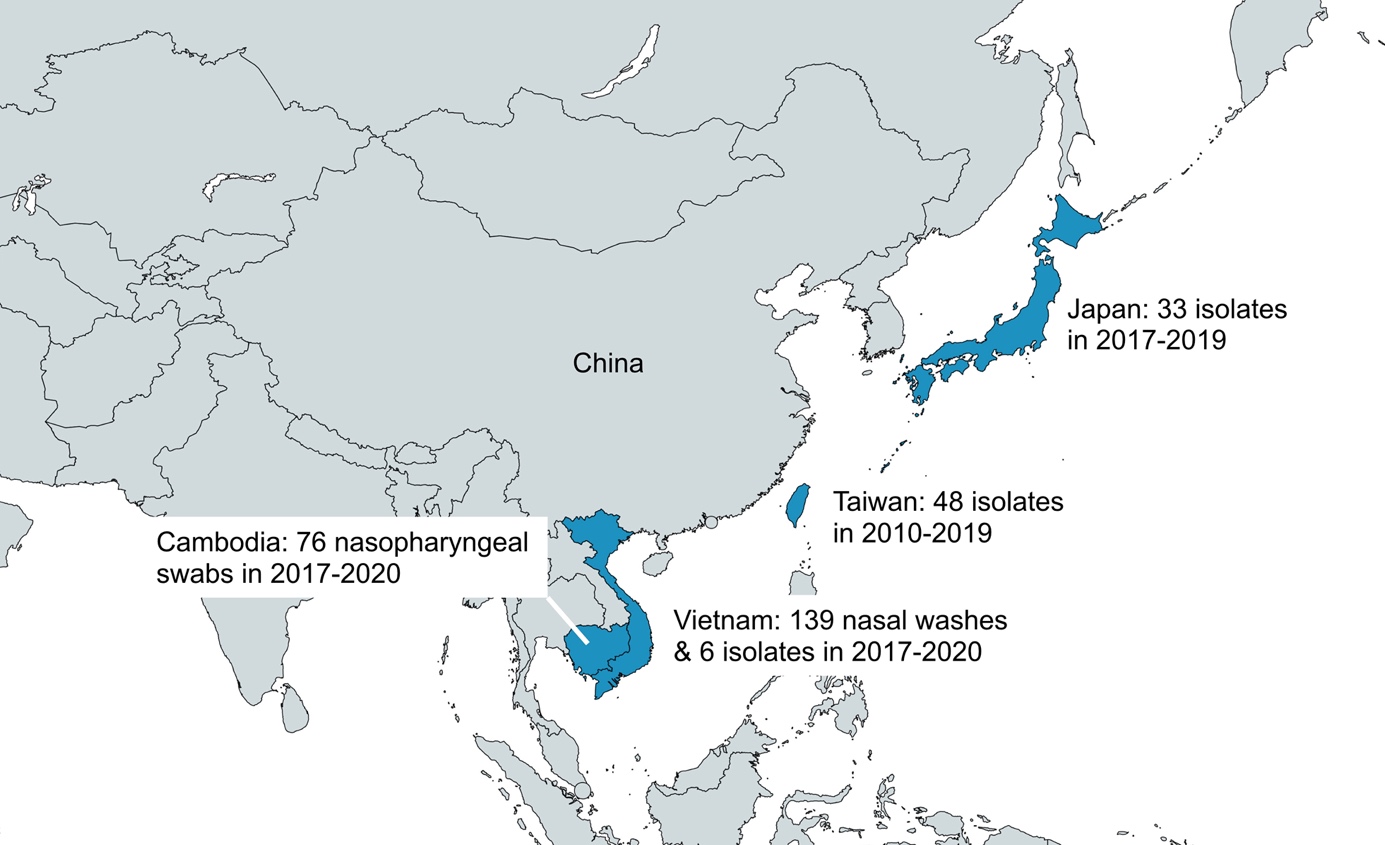


**Supplementary Figure.** Summary of clinical specimens and isolates used in this study.

Supplement: Supplementary file 1 [file mmc1.docx]
